# Supplementary figures and images for: Bestrophin-4 relays HES4 and interacts with TWIST1 to suppress epithelial-to-mesenchymal transition in colorectal cancer cells
Source: eLife. 2024 Dec 19;12:RP88879. doi: 10.7554/eLife.88879 (PMC11658771; doi:10.7554/eLife.88879)

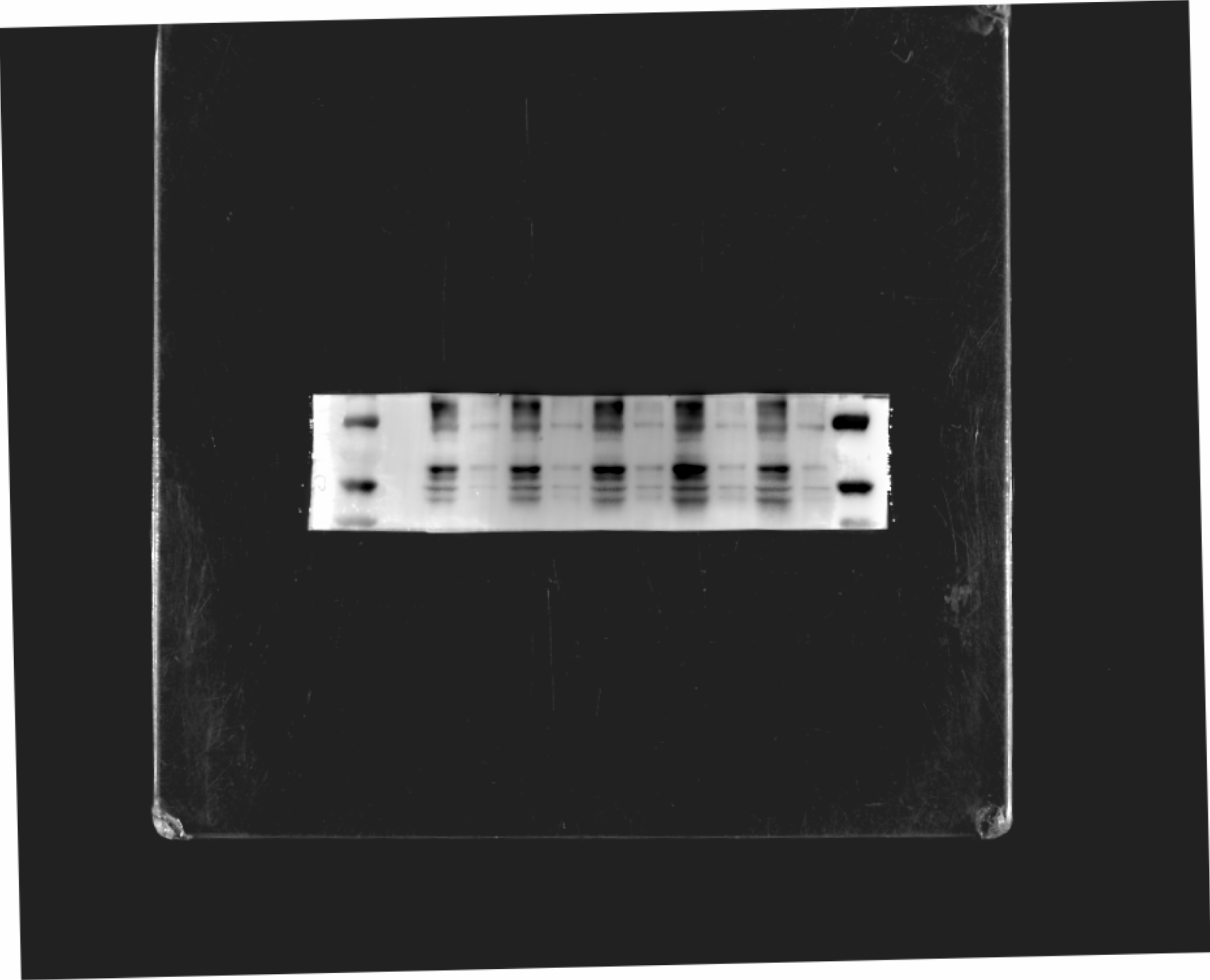

Supplement: Figure 5—source data 1. [file elife-88879-fig5-data1.zip › Fig5E-source data 1/fig5-E-BEST4 Human.tif]

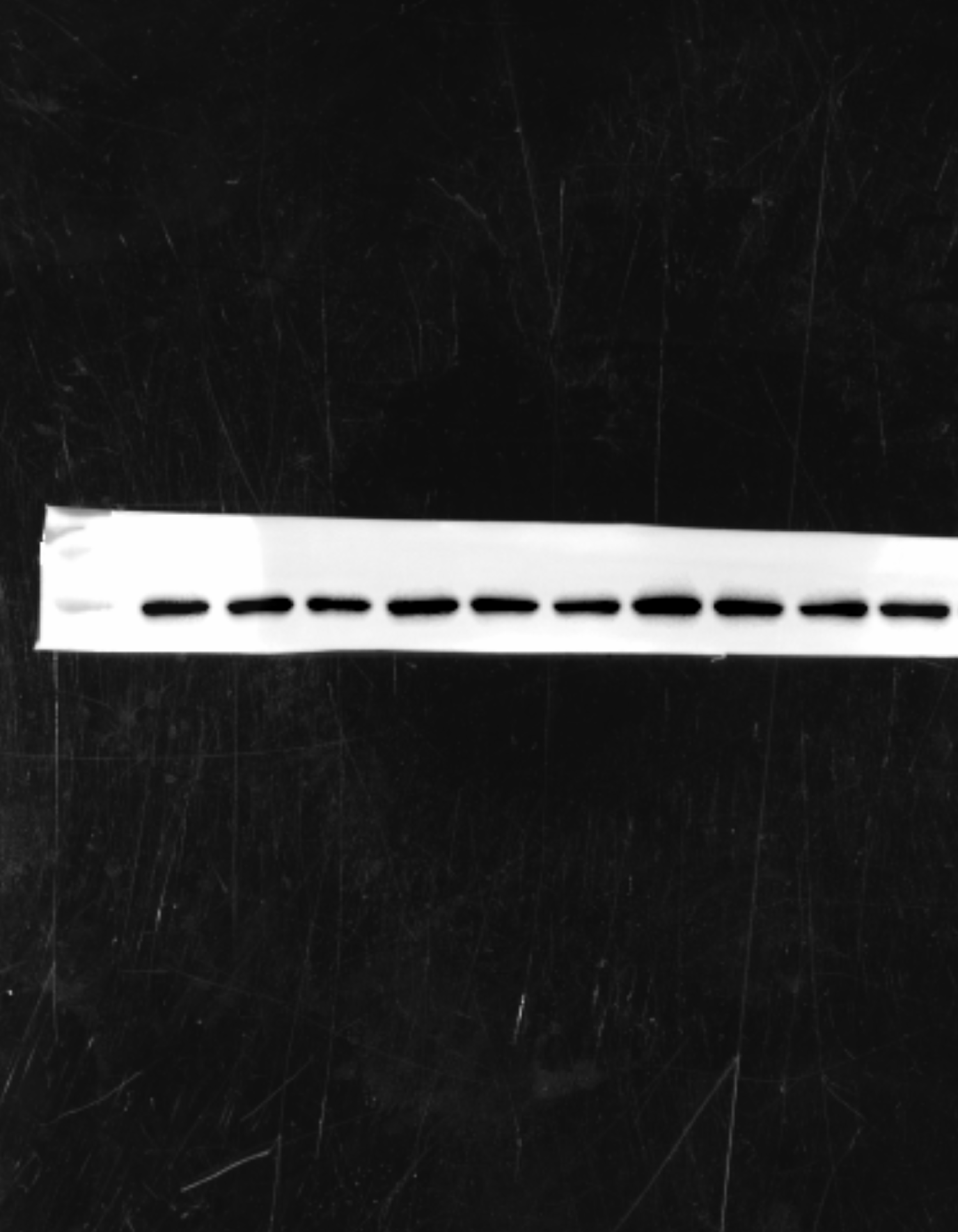

Supplement: Figure 5—source data 1. [file elife-88879-fig5-data1.zip › Fig5E-source data 1/fig5-E-α-Tublin Human.tif]

Fig 5E

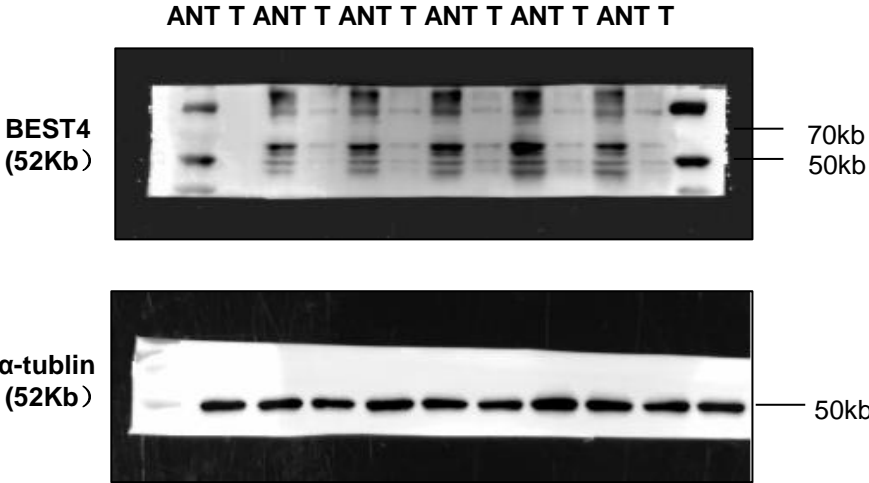

Supplement: Figure 5—source data 2. [file elife-88879-fig5-data2.zip › Fig5E-source data 2.pdf]
