# Supplementary material for: Bestrophin-4 relays HES4 and interacts with TWIST1 to suppress epithelial-to-mesenchymal transition in colorectal cancer cells
Source: eLife. 2024 Dec 19;12:RP88879. doi: 10.7554/eLife.88879 (PMC11658771; doi:10.7554/eLife.88879)
Supplement: Supplementary file 1. — The target sequence of short hairpin RNAs (shRNAs), RNAs silencing (siRNAs), and primers used in this study. [file elife-88879-supp1.docx]

**Table S1. The target sequence of shRNAs, siRNAs and primers used in this study.**

| **NAME** | **5’-3’ sequence** |
| --- | --- |
| *HES4*-shRNA 01 | 5’-CCACGAGTGTCTGGCGGAGGT-3’ |
| *HES4*-shRNA 02 | 5’-ACATCCTGGAGATGACCGTGA-3’ |
| *BEST4*-siRNA 01 | 5’-CAAGTGCAGCATGCATTCCA-3’ |
| *BEST4*-siRNA 02 | 5’-ATCACGGAAGGTCTTGTCAAA-3’ |
| *BEST4*-siRNA 04 | 5’-TACCCTGGTAACCTTACTCAA-3’ |
| *BEST4*-siRNA 05 | 5’-ACGGGCGAATACGTGACGATA-3 |
| *BEST4* | Forward: 5’-CATCACCTACCGGCTG CT-3’  Reverse: 5’-GCTGATGTGCGTCATCTCGG-3’ |
| CDH1 | Forward: 5’-CATCACCTACCGGCTG CT-3’  Reverse: 5’-GCTGATGTGCGTCATCTCGG-3’ |
| Vimentin | Forward: 5’-AACTTCTCAGCATCCGATGAC-3’  Reverse: 5’-TTGTAGGAGTGTCGGTTGTTAAG-3’ |
| TJP1 | Forward: 5’-AGGGGCAGTGGTGGTTTTCTGTTCTTTC-3’  Reverse: 5’-GCAGAGGTCAAAGTTCAAGGCTCAAGAGG-3’ |
| TWIST1 | Forward: 5’-AGCTACGCCTTCTCGGTCT-3’  Reverse: 5’-CCTTCTCTGGAAACAATGACATC-3’ |
| *HES4* | Forward: 5’-GACCCGCAGTCCTCCAA-3’  Reverse: 5’-TCCGCCTTCTCCAGCTTC-3’ |
| GAPDH | Forward: 5’-ACTCCTCCACCTTTGACGC-3’  Reverse: 5’-GCTGTAGCCAAATTCGTTGTC-3’ |
| P1 | Forward: 5’- CTCTCTGTGGCCCAGGCTGGA-3’  Reverse: 5’-GCACCAGCTGTAGTCCCAGCT-3’ |
| P2 | Forward: 5’-GAGAGGAGTCTCACTCTTT-3’  Reverse: 5’-AATGGCGTGAACCTGGGAGG-3’ |
| P3 | Forward: 5’-GCCTCCTGAGTAGCTGGGAC-3’  Reverse: 5’-TCATGAGGTCAGGAGATCGA-3’ |
| P4 | Forward: 5’-GTGATCCCACCTCTGGGCTC -3’  Reverse: 5’-CCCTGGCGTGACCTGTCGGT-3’ |
| DHFR  5’ UTR | Forward: 5’-CTGATGTCCAGGAGGAGAAAGG-3’  Reverse: 5’-AGCCCGACAATGTCAAGGACTG-3’ |
