# Supplementary material for: Bestrophin-4 relays HES4 and interacts with TWIST1 to suppress epithelial-to-mesenchymal transition in colorectal cancer cells
Source: eLife. 2024 Dec 19;12:RP88879. doi: 10.7554/eLife.88879 (PMC11658771; doi:10.7554/eLife.88879)
Supplement: Figure 2—source data 2. [file elife-88879-fig2-data2.zip › Figure 2-source data 2/Figure 2C-source data 2.zip!/Figure 2C-source data 2.pdf]

# Fig 2C

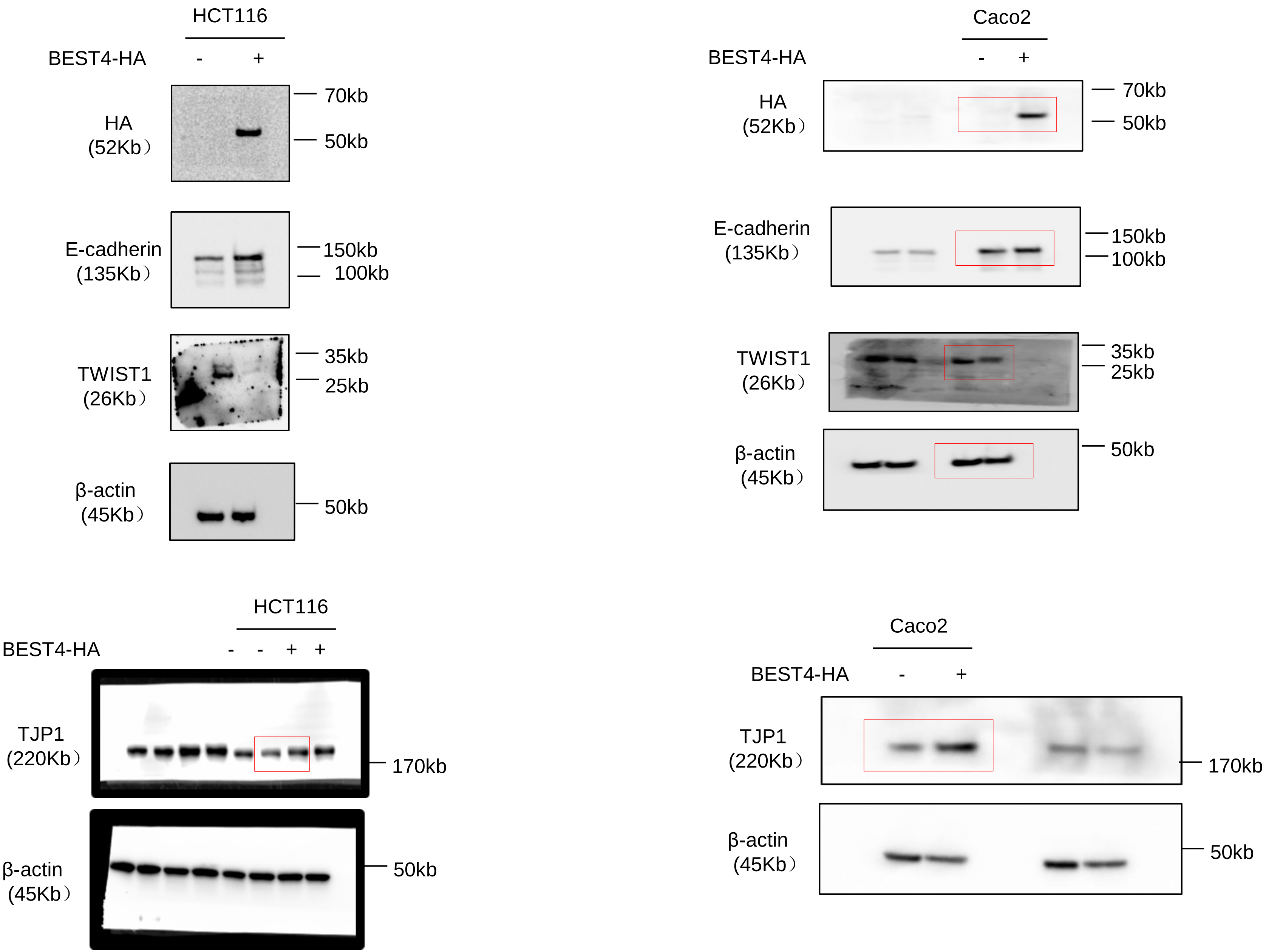
HCT116
BEST4-HA - +
HA
(52Kb)
70kb
50kb
E-cadherin
(135Kb)
150kb
100kb
TWIST1
(26Kb)
35kb
25kb
β-actin
(45Kb)
50kb
Caco2
BEST4-HA - +
HA
(52Kb)
70kb
50kb
E-cadherin
(135Kb)
150kb
100kb
TWIST1
(26Kb)
35kb
25kb
β-actin
(45Kb)
50kb
HCT116
BEST4-HA - - + +
TJP1
(220Kb)
170kb
β-actin
(45Kb)
50kb
Caco2
BEST4-HA - +
TJP1
(220Kb)
170kb
β-actin
(45Kb)
50kb
