# Supplementary material for: Bestrophin-4 relays HES4 and interacts with TWIST1 to suppress epithelial-to-mesenchymal transition in colorectal cancer cells
Source: eLife. 2024 Dec 19;12:RP88879. doi: 10.7554/eLife.88879 (PMC11658771; doi:10.7554/eLife.88879)
Supplement: Figure 2—source data 2. [file elife-88879-fig2-data2.zip › Figure 2-source data 2/Figure 2D-source data 2.zip!/Figure 2D-source data 2.pdf]

Fig 2D

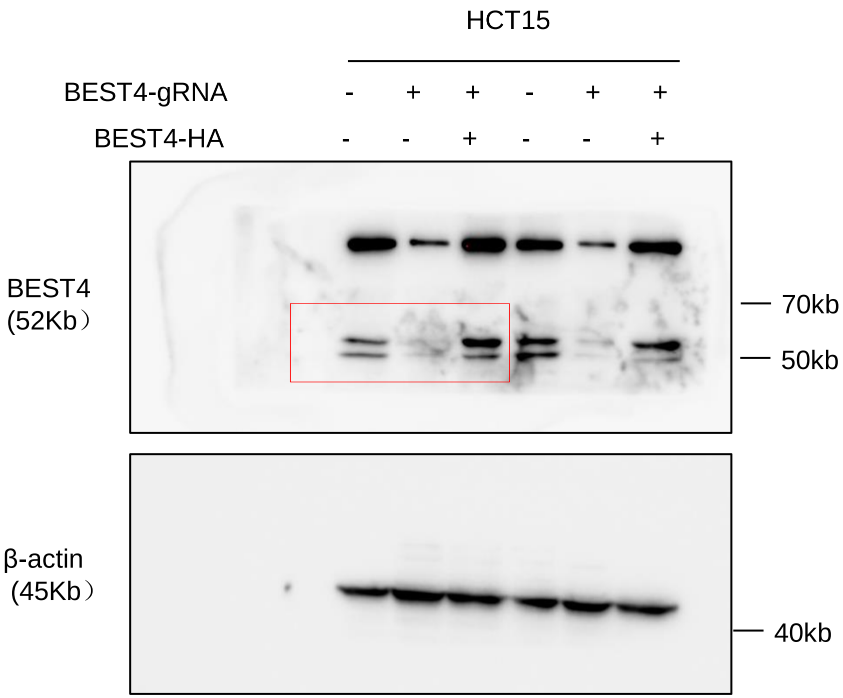
HCT15
BEST4-gRNA - + + - + +
BEST4-HA - - + - - +
BEST4 (52Kb)
70kb
50kb
β-actin (45Kb)
40kb

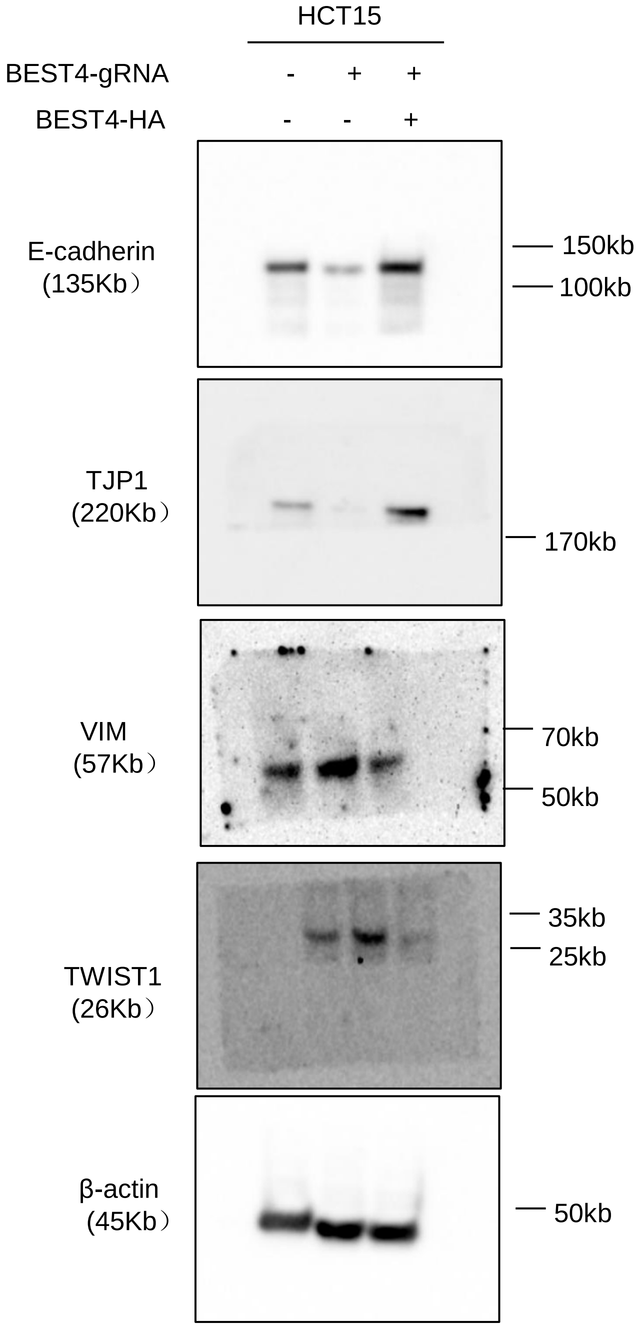
HCT15
BEST4-gRNA - + +
BEST4-HA - - +
E-cadherin (135Kb)
150kb
100kb
TJP1 (220Kb)
170kb
VIM (57Kb)
70kb
50kb
TWIST1 (26Kb)
35kb
25kb
β-actin (45Kb)
50kb
