# Supplementary material for: Bestrophin-4 relays HES4 and interacts with TWIST1 to suppress epithelial-to-mesenchymal transition in colorectal cancer cells
Source: eLife. 2024 Dec 19;12:RP88879. doi: 10.7554/eLife.88879 (PMC11658771; doi:10.7554/eLife.88879)
Supplement: Figure 2—source data 2. [file elife-88879-fig2-data2.zip › Figure 2-source data 2/Figure 2I-source data 2.zip!/Figure 2I-source data 2.pdf]

Fig 2I

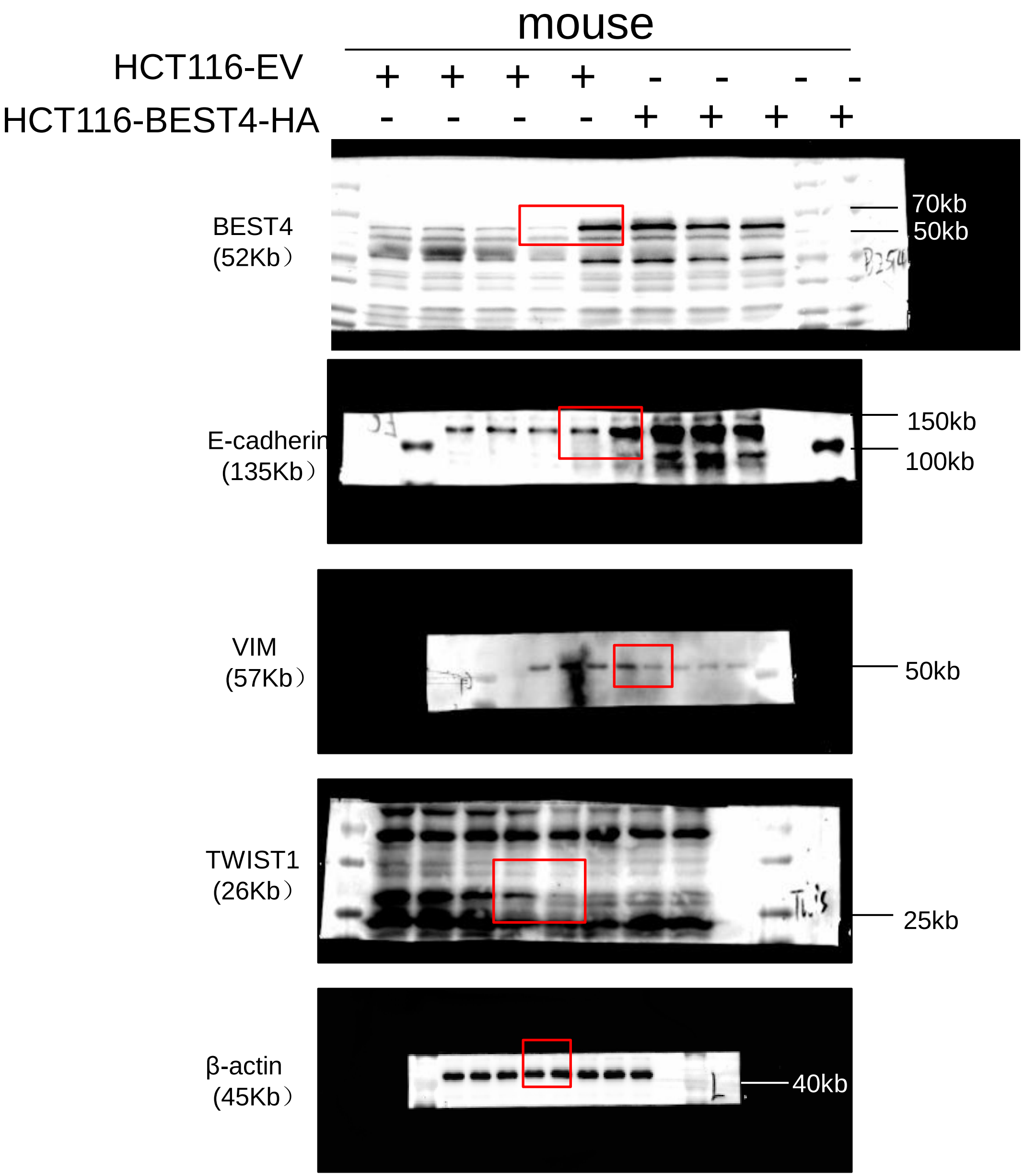
mouse
HCT116-EV + + + + - - - -
HCT116-BEST4-HA - - - - + + + +
BEST4
(52Kb）
70kb
50kb
E-cadherin
(135Kb）
150kb
100kb
VIM
(57Kb）
50kb
TWIST1
(26Kb）
25kb
β-actin
(45Kb）
40kb
