# Supplementary material for: Bestrophin-4 relays HES4 and interacts with TWIST1 to suppress epithelial-to-mesenchymal transition in colorectal cancer cells
Source: eLife. 2024 Dec 19;12:RP88879. doi: 10.7554/eLife.88879 (PMC11658771; doi:10.7554/eLife.88879)
Supplement: Figure 3—source data 2. [file elife-88879-fig3-data2.zip › Figure3-source data 2/Fig3B-source data 2.zip!/Fig3B-source data 2.pdf]

Fig 3B

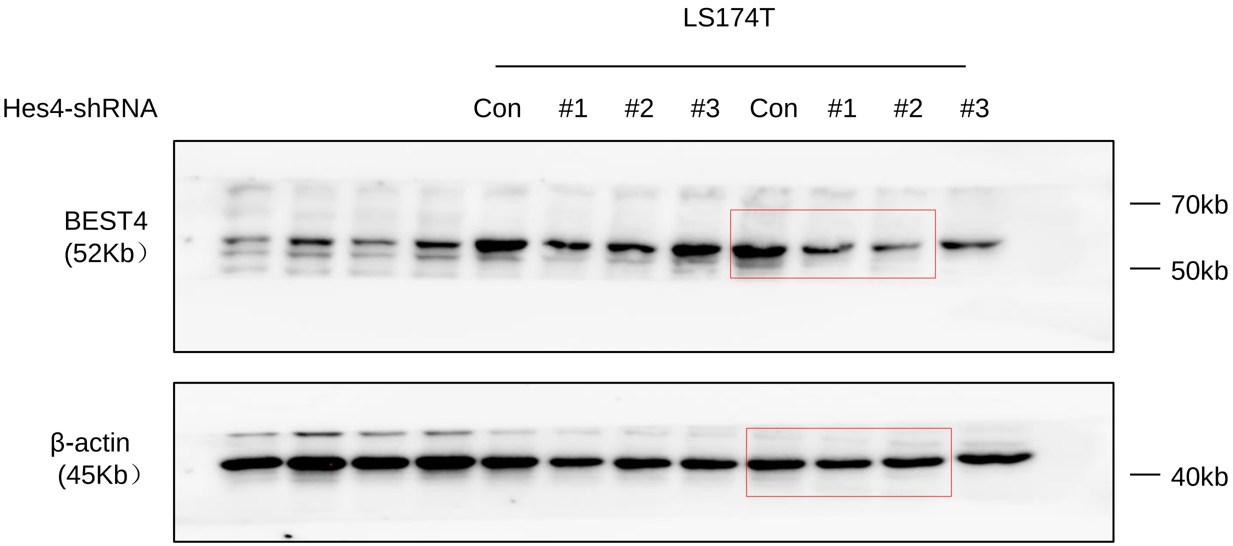
LS174T
Hes4-shRNA
Con
#1
#2
#3
Con
#1
#2
#3
BEST4
(52Kb）
β-actin
(45Kb）
70kb
50kb
40kb
