# Supplementary material for: Bestrophin-4 relays HES4 and interacts with TWIST1 to suppress epithelial-to-mesenchymal transition in colorectal cancer cells
Source: eLife. 2024 Dec 19;12:RP88879. doi: 10.7554/eLife.88879 (PMC11658771; doi:10.7554/eLife.88879)
Supplement: Figure 3—source data 2. [file elife-88879-fig3-data2.zip › Figure3-source data 2/Fig3F-source data 2.zip!/Fig3F-source data 2.pdf]

Fig 3F

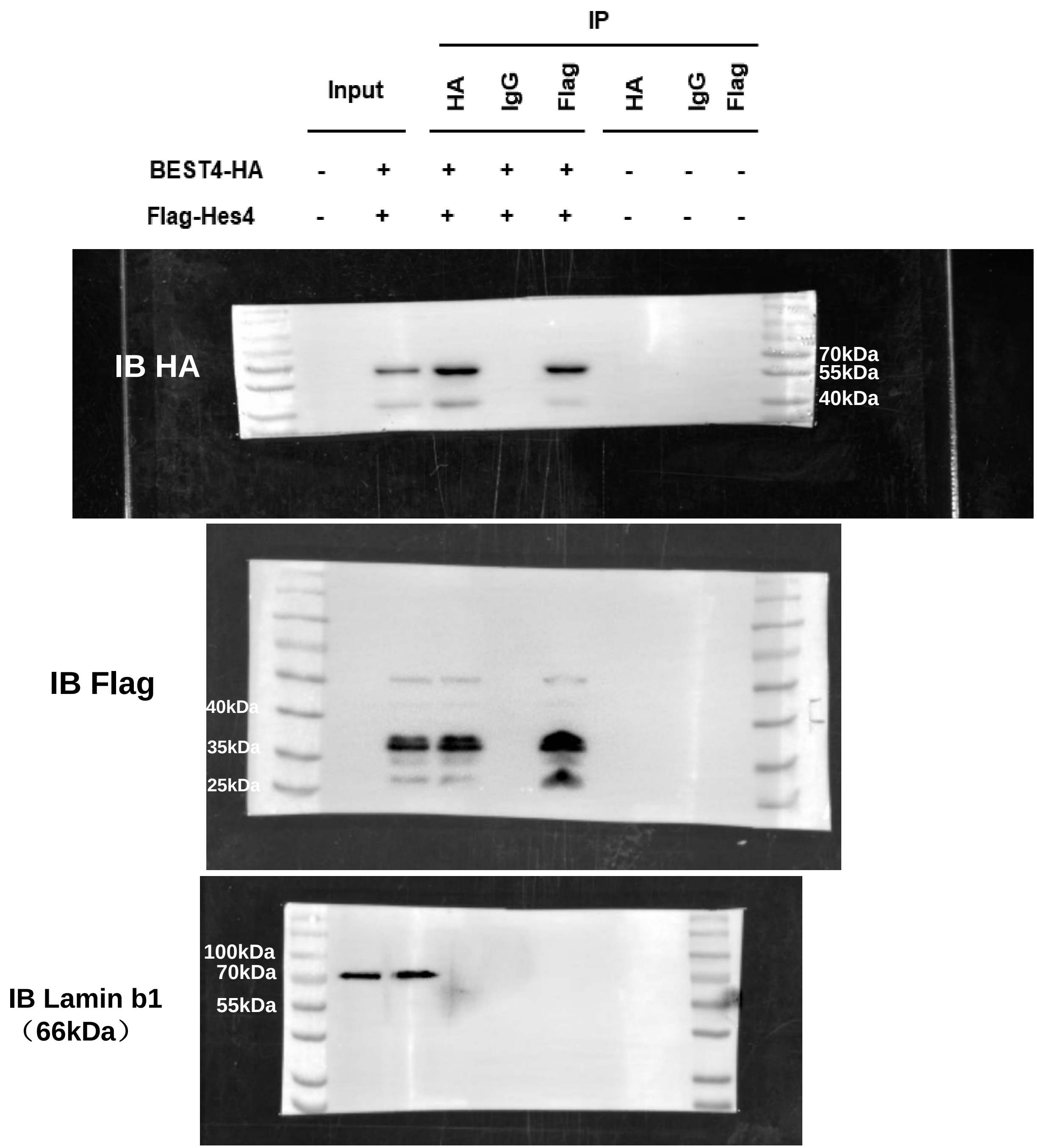
IP
Input
HA
IgG
Flag
HA
IgG
Flag
BEST4-HA - + + + + - - -
Flag-Hes4 - + + + + - - -
IB HA
70kDa
55kDa
40kDa
IB Flag
40kDa
35kDa
25kDa
IB Lamin b1
（66kDa）
100kDa
70kDa
55kDa
