# Supplementary material for: Bestrophin-4 relays HES4 and interacts with TWIST1 to suppress epithelial-to-mesenchymal transition in colorectal cancer cells
Source: eLife. 2024 Dec 19;12:RP88879. doi: 10.7554/eLife.88879 (PMC11658771; doi:10.7554/eLife.88879)
Supplement: Figure 4—source data 2. [file elife-88879-fig4-data2.zip › Figure 4-source data 2/Fig4E-source data 2.zip!/Fig4E-source data 2.pdf]

Fig 4E

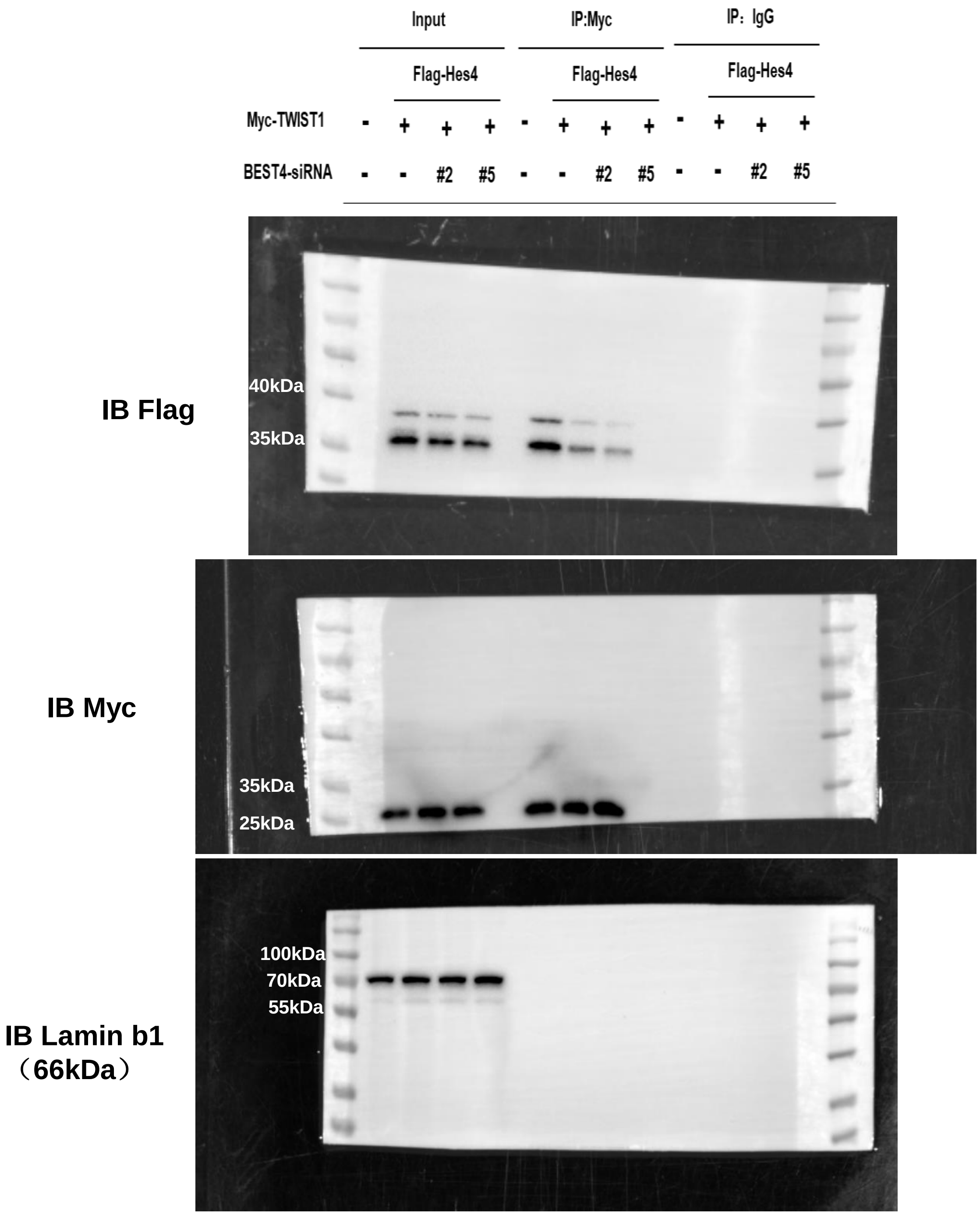

Input
IP:Myc
IP：IgG
Flag-Hes4
Flag-Hes4
Flag-Hes4
Myc-TWIST1 - + + + - + + + - + + +
BEST4-siRNA - - #2 #5 - - #2 #5 - - #2 #5
IB Flag
40kDa
35kDa
IB Myc
35kDa
25kDa
IB Lamin b1
（66kDa）
100kDa
70kDa
55kDa
