# Supplementary material for: Bestrophin-4 relays HES4 and interacts with TWIST1 to suppress epithelial-to-mesenchymal transition in colorectal cancer cells
Source: eLife. 2024 Dec 19;12:RP88879. doi: 10.7554/eLife.88879 (PMC11658771; doi:10.7554/eLife.88879)
Supplement: Figure 4—source data 2. [file elife-88879-fig4-data2.zip › Figure 4-source data 2/Fig4G-source data 2.zip!/Fig4G-source data 2.pdf]

Fig 4G

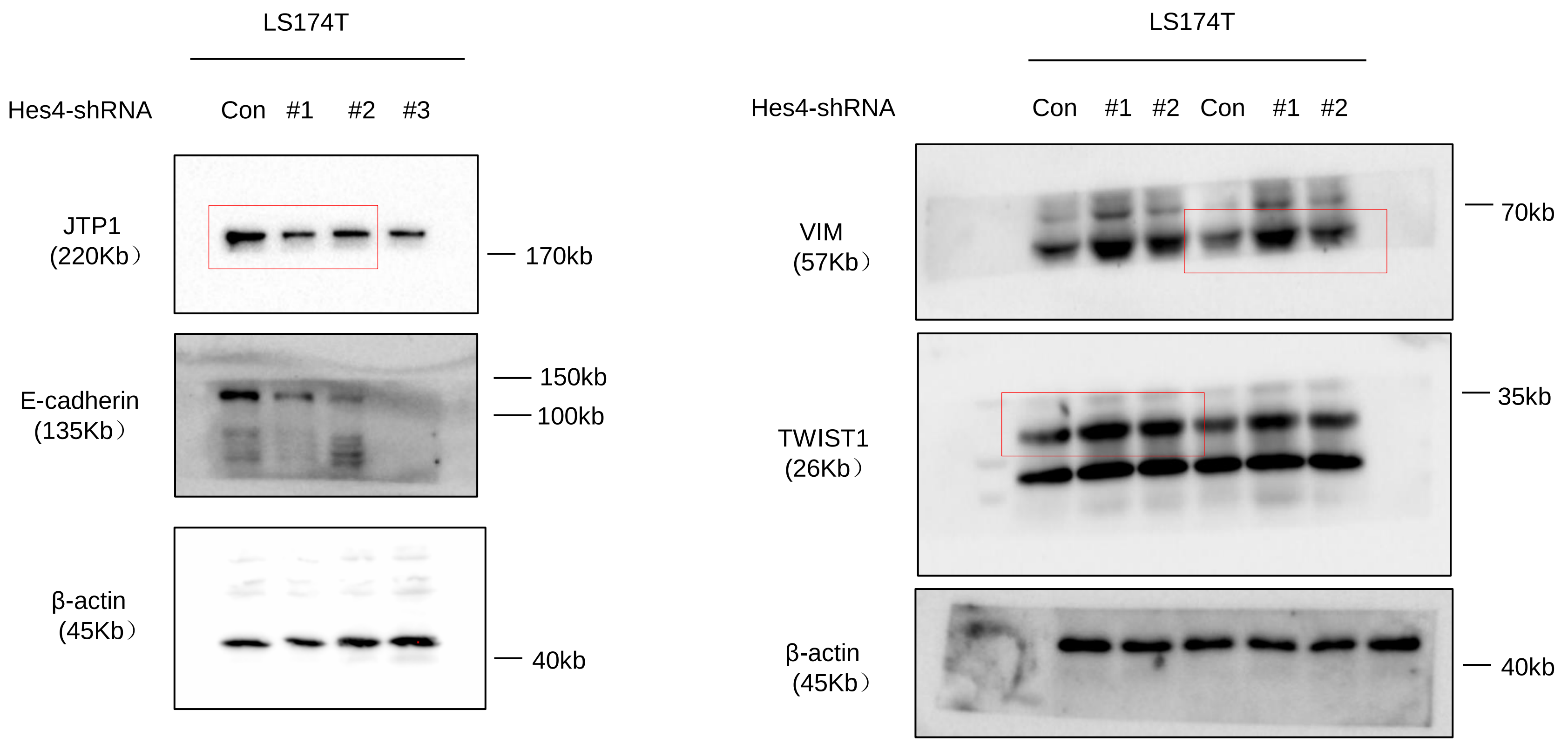

LS174T
Hes4-shRNA
Con #1 #2 #3
JTP1
(220Kb)
170kb
E-cadherin
(135Kb)
150kb
100kb
β-actin
(45Kb)
40kb
LS174T
Hes4-shRNA
Con #1 #2 Con #1 #2
VIM
(57Kb)
70kb
TWIST1
(26Kb)
35kb
β-actin
(45Kb)
40kb
