# Supplementary material for: Bestrophin-4 relays HES4 and interacts with TWIST1 to suppress epithelial-to-mesenchymal transition in colorectal cancer cells
Source: eLife. 2024 Dec 19;12:RP88879. doi: 10.7554/eLife.88879 (PMC11658771; doi:10.7554/eLife.88879)
Supplement: Figure 4—source data 2. [file elife-88879-fig4-data2.zip › Figure 4-source data 2/Fig4I-source data 2.zip!/Fig4I-source data 2.pdf]

Fig 4I

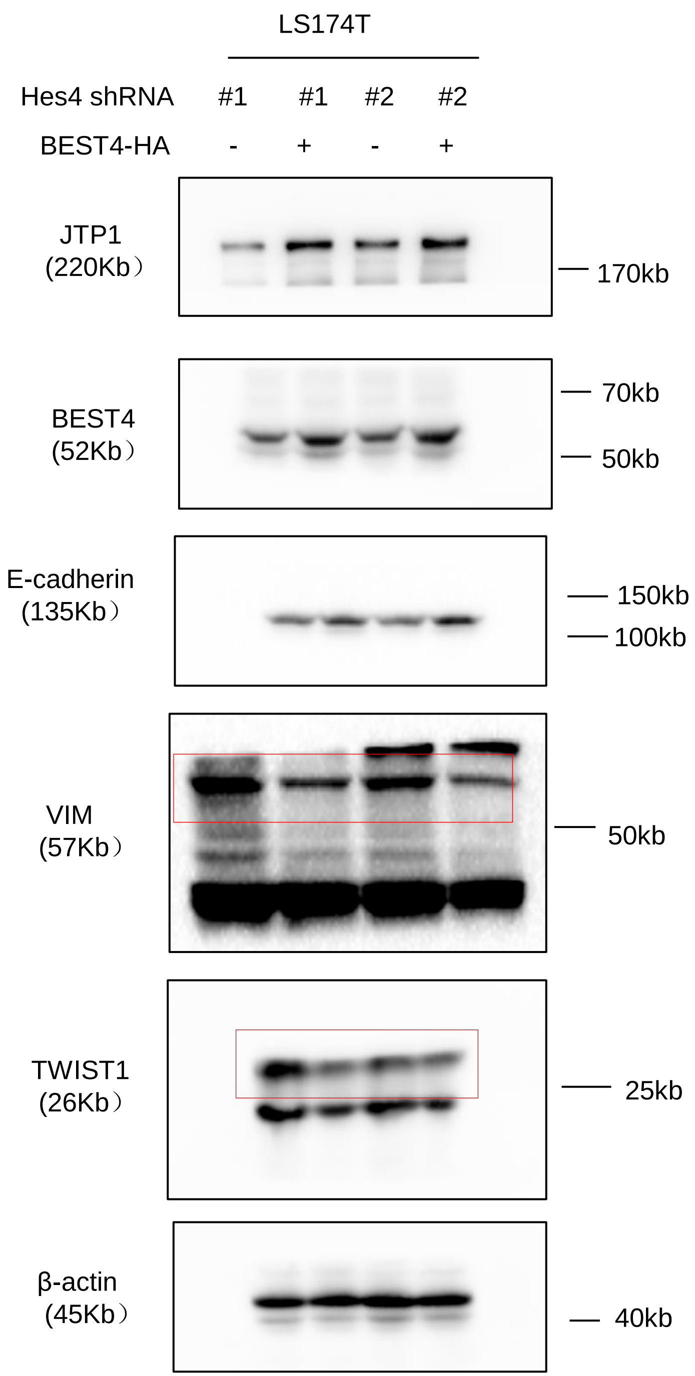

LS174T
Hes4 shRNA #1 #1 #2 #2
BEST4-HA - + - +
JTP1
(220Kb）
170kb
BEST4
(52Kb）
70kb
50kb
E-cadherin
(135Kb）
150kb
100kb
VIM
(57Kb）
50kb
TWIST1
(26Kb）
25kb
β-actin
(45Kb）
40kb
