# Supplementary material for: Bestrophin-4 relays HES4 and interacts with TWIST1 to suppress epithelial-to-mesenchymal transition in colorectal cancer cells
Source: eLife. 2024 Dec 19;12:RP88879. doi: 10.7554/eLife.88879 (PMC11658771; doi:10.7554/eLife.88879)

Figure 4-supplemental figure 1B

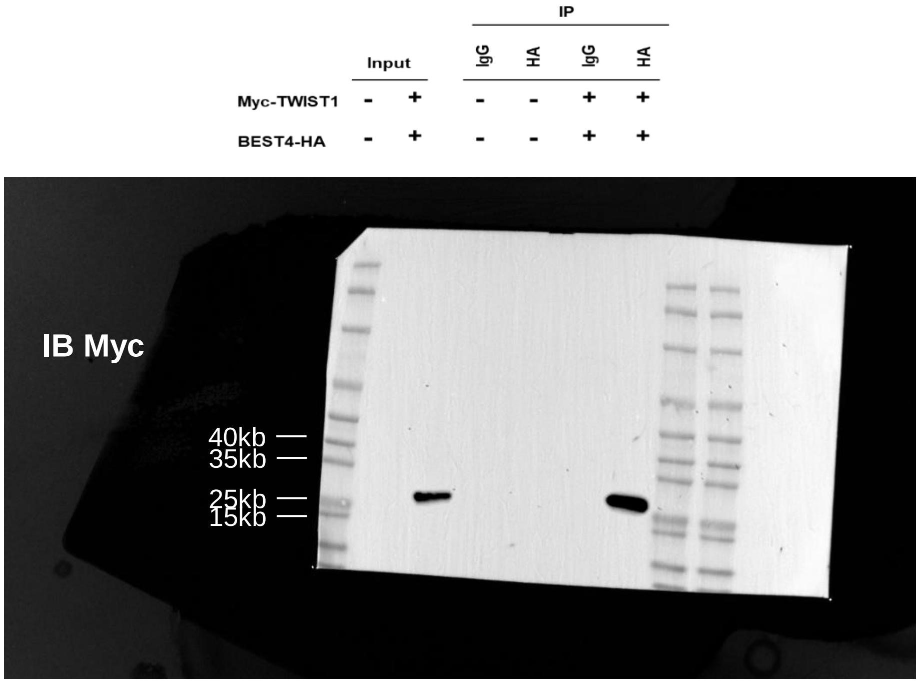

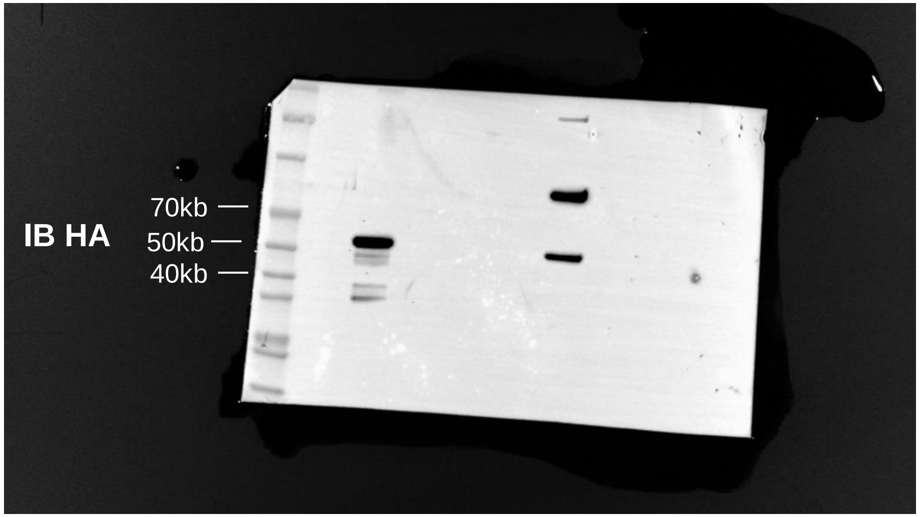

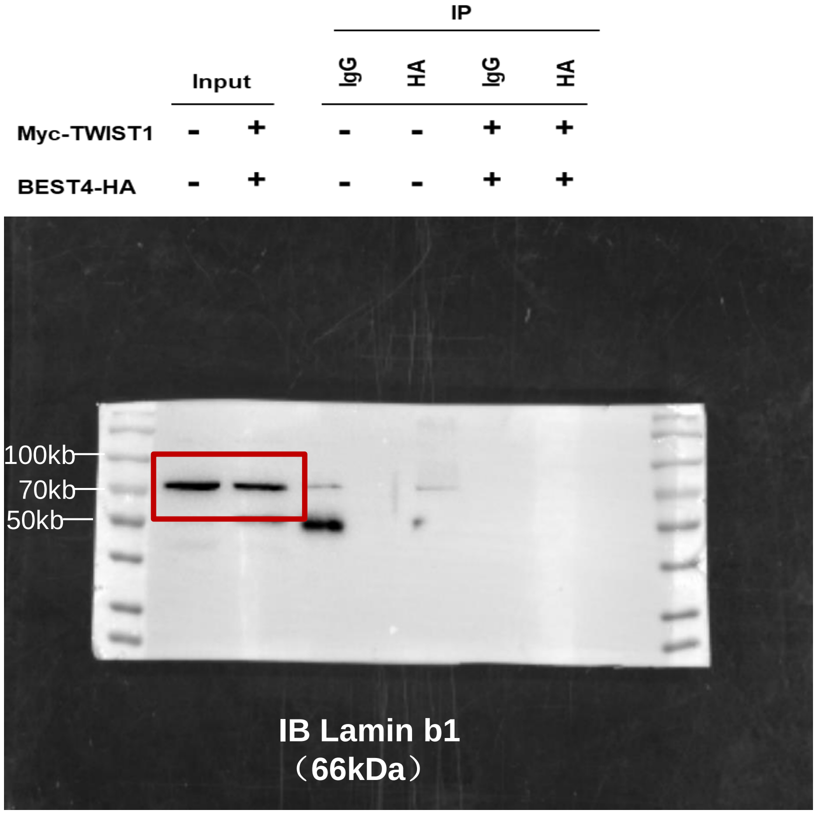

Supplement: Figure 4—figure supplement 1—source data 2. [file elife-88879-fig4-figsupp1-data2.zip › Figure 4-supplemental figure 1-source data 2/Figure 4-supplemental figure 1B-source data 2.zip!/Figure 4-supplemental figure 1B-source data 2.pdf]
